# Genomic Interaction Profiles in Breast Cancer Reveal Altered Chromatin Architecture

**DOI:** 10.1371/journal.pone.0073974

**Published:** 2013-09-03

**Authors:** Michael J. Zeitz, Ferhat Ay, Julia D. Heidmann, Paula L. Lerner, William S. Noble, Brandon N. Steelman, Andrew R. Hoffman

**Affiliations:** 1 Department of Medicine, Veterans Affairs Palo Alto Health Care System, Stanford University Medical School, Palo Alto, California, United States of America; 2 Department of Genome Sciences, University of Washington, Seattle, Washington, United States of America; 3 Department of Computer Science and Engineering, University of Washington, Seattle, Washington, United States of America; Virginia Commonwealth University, United States of America

## Abstract

Gene transcription can be regulated by remote enhancer regions through chromosome looping either in *cis* or in *trans*. Cancer cells are characterized by wholesale changes in long-range gene interactions, but the role that these long-range interactions play in cancer progression and metastasis is not well understood. In this study, we used *IGFBP3*, a gene involved in breast cancer pathogenesis, as bait in a 4C-seq experiment comparing normal breast cells (HMEC) with two breast cancer cell lines (MCF7, an ER positive cell line, and MDA-MB-231, a triple negative cell line). The *IGFBP3* long-range interaction profile was substantially altered in breast cancer. Many interactions seen in normal breast cells are lost and novel interactions appear in cancer lines. We found that in HMEC, the breast carcinoma amplified sequence gene family (*BCAS*) 1–4 were among the top 10 most significantly enriched regions of interaction with *IGFBP3.* 3D-FISH analysis indicated that the translocation-prone *BCAS* genes, which are located on chromosomes 1, 17, and 20, are in close physical proximity with *IGFBP3* and each other in normal breast cells. We also found that epidermal growth factor receptor (*EGFR),* a gene implicated in tumorigenesis, interacts significantly with *IGFBP3* and that this interaction may play a role in their regulation. Breakpoint analysis suggests that when an *IGFBP3* interacting region undergoes a translocation an additional interaction detectable by 4C is gained. Overall, our data from multiple lines of evidence suggest an important role for long-range chromosomal interactions in the pathogenesis of cancer.

## Introduction

It is now widely recognized that the spatial organization of the genome and not only its linear sequence is essential for normal genome function [1]. Recent breakthroughs combining high-throughput DNA sequencing and molecular assays have revolutionized our understanding of chromatin organization [2], [3], [4]. Three-dimensional chromatin structure is important in the regulation of transcription [5], and in the control of epigenetic states (including the regulation of imprinted genes) by means of chromosome looping between distant regulatory regions on the same or on different chromosomes [6], [7]. Dynamic, long-range interactions have been observed to regulate gene expression, contribute to the developmental processes of T cell differentiation and X-inactivation, and may play a role in tumorigenesis [7], [8], [9], [10], [11]. The interchromosomal interaction between the *Ifng* promoter on chromosome 10 and the T_H_2 cytokine gene locus on chromosome 11 in naive T cells maintains both loci in a configuration poised for rapid transcription and is thought to facilitate the developmental choice between T_H_1 or T_H_2 cells [8]. Transient homologous pairing of X-inactivation centers early in development is crucial for correct X chromosome dosage compensation in mammalian females [9], [10]. We have shown that *Igf2* on chromosome 7 interacts with the *Wsb1/Nf1* locus on chromosome 11, and disruption of this interaction results in decreased expression of *Wsb1* and *Nf1* [7]. We also observed a substantial alteration in chromatin structure within human cancers that have lost *IGF2* imprinting, resulting in a striking loss of long-range interactions across the *IGF2/H19* locus [11]. These studies indicate that a better understanding of intricate 3D chromatin organization is crucial to understanding human diseases, particularly cancer, in which genomic instability and dysregulation are widespread.

Breast cancer is a complex disease that involves alterations in both genetic and epigenetic factors [12], [13], [14]. While numerous genetic mutations, translocations and aberrant DNA methylation have been reported in breast cancer, the role of long-range interactions during cancer progression remains elusive. Recent evidence suggests that genome organization is altered early in breast tumorigenesis [15]. Cancer-related genes were observed to change their radial positions in a cell culture model of early breast tumor development [15]. Changes in radial position of cancer-related genes were also observed in breast tumor tissue samples, and were not caused by genomic instability [16].

Insulin-like growth factor binding protein 3 (*IGFBP3)* has been implicated in breast cancer pathogenesis [17], [18], [19], [20], [21]. IGFBP3 modulates cell growth and survival by binding to insulin-like growth factors I and II, and regulating their bioavailability [22]. IGFBP3 has also been proposed to function independently of IGF-I or IGF-II and act as a growth modulator [23], [24], [25]. While correlations between serum levels of IGFBP3 and breast cancer have yielded contradictory results [19], [20], [21], [26], increased levels of IGFBP3 in breast cancer tissue is correlated with a worse prognosis and poor clinical features [17], [18].

Dysregulation of *IGFBP3* expression and hypermethylation of its promoter have been observed in many cancers [27]. Increased *IGFBP3* expression has been shown to enhance survival of breast cancer cells exposed to environmental stress [28]. Alternatively, a mouse model of prostate cancer crossed with a knockout of *Igfbp3* displayed significant increase in metastasis in double mutant animals. *In vitro* assays of prostate cell lines derived from these mouse lines also indicated a more aggressive cancer phenotype in IGFBP3 deficient cells [29]. We sought to explore global differences of *IGFBP3* long-range interaction profiles between normal breast cells and breast cancer cell lines. We hypothesized that cancer-related changes in *IGFBP3* regulation and epigenetic modification might coincide with altered spatial positioning and long-range DNA interactions contributing to breast cancer pathogenesis. We therefore used the *IGFBP3* enhancer as bait in circular chromosome conformation capture with high-throughput sequencing (4C-seq) in normal human mammary epithelial cells (HMEC) and two breast cancer cell lines, MCF7 and MDA-MB-231. MCF7 and MDA-MB-231 represent distinct breast cancer subtypes. MCF7 is a human breast adenocarcinoma cell line positive for estrogen receptor alpha, and MDA-MB-231 is a human breast carcinoma cell line negative for estrogen and progesterone receptors as well as *HER2*. The *IGFBP3* promoter displays hypermethylation, and there is reduced *IGFBP3* expression in MCF7, while in MDA-MB-231, the promoter is relatively hypomethylated, and *IGFBP3* is over-expressed compared to HMEC.

In this study, we examined *IGFBP3* long-range interactions and show that the three-dimensional structure of the genome changes dramatically in breast cancer. Our data suggest a possible role for long-range chromatin interactions in the pathogenesis of breast cancer as well as in the formation of translocations often seen in malignant cells.

## Results

### Expression of IGFBP3 is Downregulated in MCF7, but Upregulated in MDA-MB-231 Relative to HMEC

**Figure 1:**
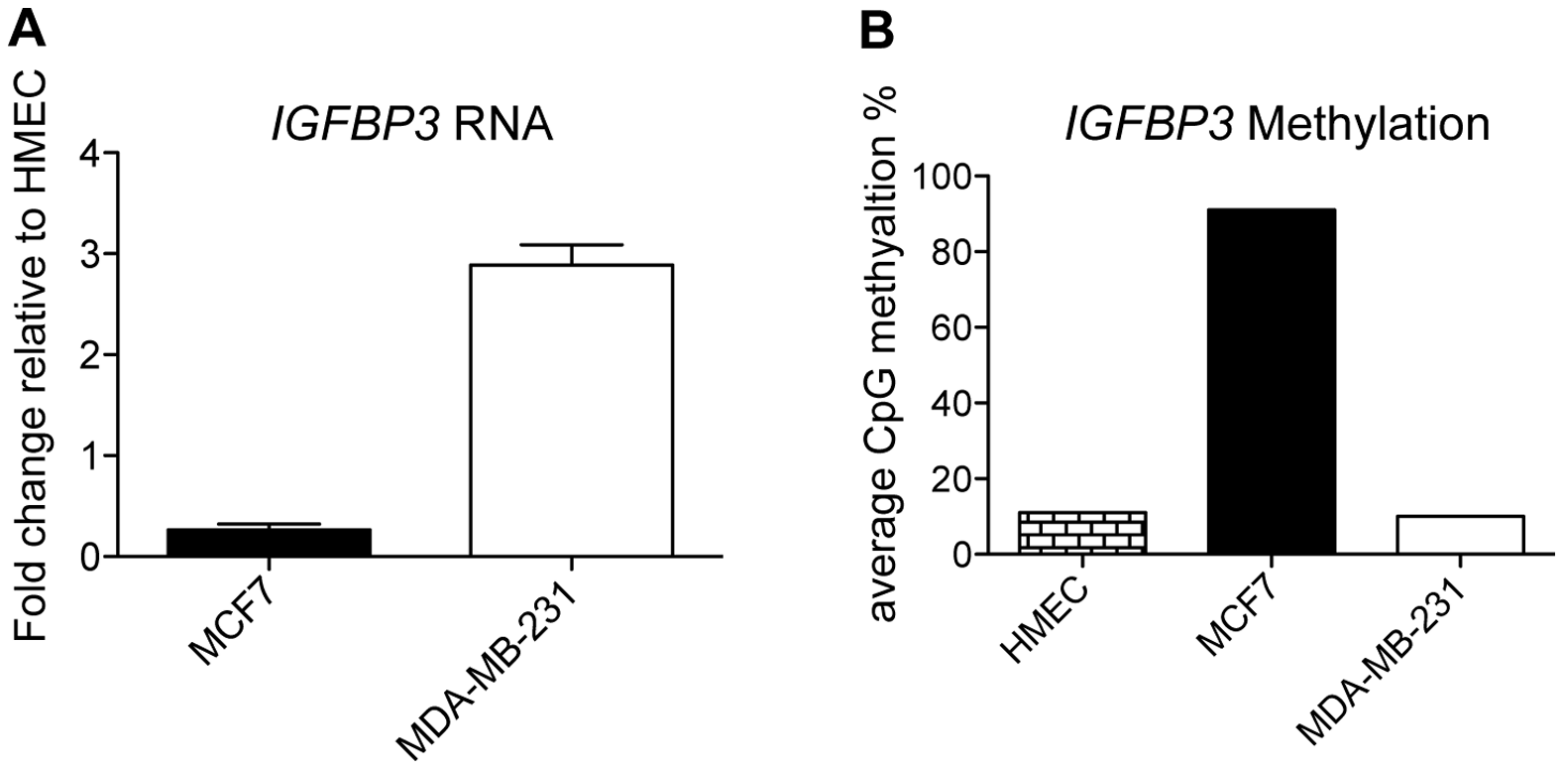
Expression and methylation status of *IGFBP3*. A, qRT-PCR: RNA levels of *IGFBP3* were measured in MCF7, MDA-MB-231 and HMEC cells. Expression in cancer lines was plotted as fold change relative to HMEC. Data represent the SEM of three independent biological replicates. B, Percent methylation of CpG nucleotides in the *IGFBP3* promoter in HMEC, MCF7 and MDA-MB-231. Bars represent the average percent methylation of 4 positions in the *IGFBP3* promoter.

To better understand the role of *IGFBP3* in breast cancer, we analyzed its expression in primary breast cells, the estrogen receptor alpha (ERα) positive breast cancer cell line MCF7, and the triple-negative breast cancer cell line MDA-MB-231. *IGFBP3* expression was increased nearly 3-fold in MDA-MB-231, and reduced 3.8-fold in MCF7, relative to HMEC (Figure 1A). To evaluate whether DNA methylation correlated with the changes in expression, we examined the methylation status of the *IGFBP3* promoter by bisulfite pyrosequencing. The *IGFBP3* promoter was hypermethylated (91% CpG methylation) in MCF7 compared with 11% and 10% CpG methylation in HMEC and MDA-MB-231, respectively (Figure 1B).

### EGFR Interacts Significantly with IGFBP3

**Figure 2:**
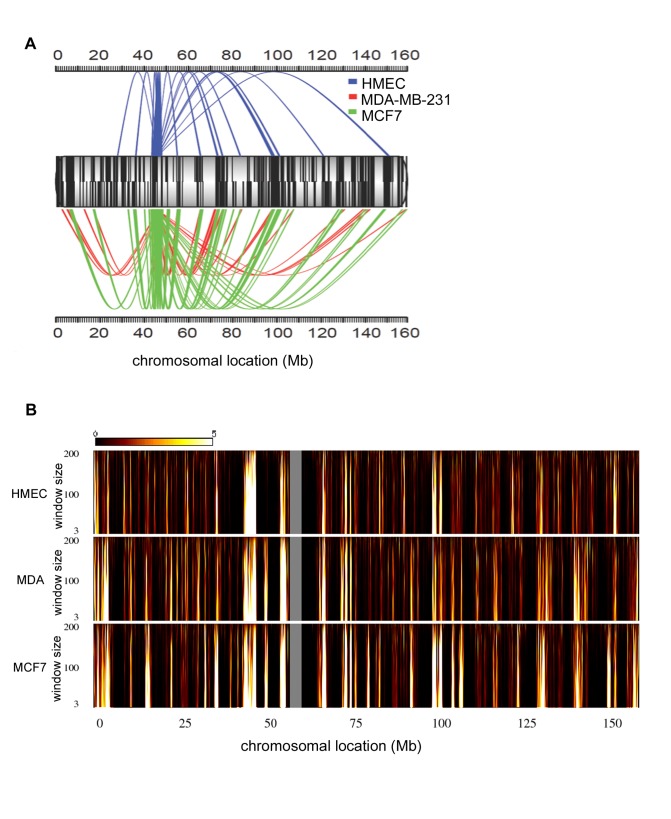
Intrachromosomal interaction profile of *IGFBP3*. A, Spider plot showing the significant long-range interactions of the *IGFBP3* enhancer across chromosome 7 for a window size of 100 consecutive restriction fragments in HMEC (blue), MDA-MB-231 (red), and MCF7 (green). Mb position is plotted. Tick marks on chromosome 7 represent gene locations with positive strand genes on top and negative strand genes on bottom. B, Domainograms illustrating the significance of intrachromosomal interactions for window sizes ranging from 3 to 200 consecutive fragments for each cell line. The color represents −log(p-value) of the calculated significance score ranging from black (not significant) to white (most significant). The gray region corresponds to the centromere of chromosome 7, which lacks HindIII cut sites.

To identify whether changes in *IGFBP3* expression and methylation were accompanied by global alteration of its long-range chromatin interactions, we performed multiplex 4C-seq in HMEC, MCF7 and MDA-MB-231. We chose as our bait a region upstream of *IGFBP3* classified as a strong enhancer in HMEC by chromatin profiling of several distinctive features, including enrichment of the enhancer marks H3K4me1 and H3K4me2 and the active regulatory H3K9ac and H3K27ac marks (Figure S1) [30]. We obtained a combined total of approximately 12 million mapped reads from the three cell lines with the majority mapping in *cis* (Table S1). The 4C-seq reads were binned into windows based on the number of mappable HindIII restriction sites, ranging from 25 to 400. Regions with a false discovery rate (FDR) below 0.01 (see Methods) were considered to be significantly interacting. The significant long-range *cis* interactions for window size 100 in HMEC, MCF7 and MDA-MB-231 are diagrammed in Figure 2A. For every window size analyzed, MCF7 contained the largest number of significant long-range intrachromosomal interactions, followed by MDA-MB-231 and HMEC. Using a window size of 100, there were a total of 16 significant *cis* long-range interactions in HMEC, 51 in MCF7 and 29 in MDA-MB-231. Of these interactions, 8 were common to all 3 cell lines, indicating a 50% conservation of all high confidence long-range interactions from HMEC (Figure 2B). Numerous novel long-range interactions were observed in each cancer cell line, and some long-range interactions found in normal cells were lost in each cancer cell line.

**Figure 3:**
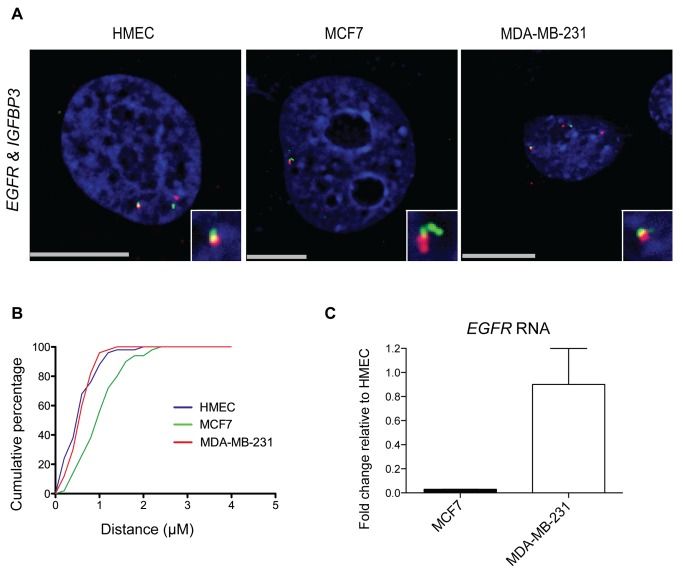
Interaction frequency of *IGFBP3* with the breast cancer related gene *EGFR* by 3D-FISH. A, 3D-FISH labeling of breast cancer related loci in HMEC, MCF7, MDA-MB-231. BAC probe combinations: *IGFBP3* (green) and *EGFR* (red) n = 50, DAPI DNA stain (blue), boxes in lower right corner contain a magnified view of each interaction. Scale bar  = 10 µm. B, Cumulative percentage of distances between *IGFBP3* and *EGFR* loci. Distances were measured between the closest two foci in each nucleus. C, qRT-PCR: RNA levels of *EGFR* measured in MCF7, MDA-MB-231 and HMEC cells. Expression in cancer lines plotted as fold change relative to HMEC. Data represent the SEM of three independent biological replicates.

Among the significant intrachromosomal interactions common to all samples, and across all window sizes, was an interaction with epidermal growth factor receptor (*EGFR*), another breast cancer related gene. *EGFR* is located approximately 9 Mb from *IGFBP3* on chromosome 7. To examine this long-range interaction in more detail, we labeled gene pairs *EGFR* and *IGFBP3* by 3D-FISH in HMEC and breast cancer cell lines MCF7 and MDA-MB-231 (Figure 3A). To quantitate differences in interaction frequencies at the cellular level, we measured the center-to-center distances between the closest pairs of labeled foci. In 88% of HMEC nuclei counted, *EGFR* and *IGFBP3* were within 1 micron of each other, indicating frequent interactions (Figure 3B). This interaction frequency was only 56% in MCF7 nuclei, but was 96% in MDA-MB-231 nuclei. To assess whether differences in spatial positioning were accompanied by changes in expression, we measured RNA levels of *EGFR* in HMEC, MCF7 and MDA-MB-231 by qRT-PCR (Figure 3C). Relative to HMEC, *EGFR* expression was unchanged in MDA-MB-231, yet it was reduced 35-fold to nearly undetectable levels in MCF7 cells. In contrast to *IGFBP3,* the expression change in *EGFR* was not accompanied by a change in CpG methylation in the *EGFR* promoter among the three cell lines (data not shown). This suggests the difference in *EGFR* expression could be driven in part by chromatin architecture rather than methylation. In MCF7, the reduction in long-range interaction frequency with *EGFR* provides the opportunity for *IGFBP3* to form additional contacts. This may partially explain the gain of 35 unique intrachromosomal interactions in MCF7 cells compared to HMEC.

### Interchromosomal Rearrangements Involving IGFBP3 Interacting Regions Facilitate an Increase in Long-Range Interactions in MCF7

**Figure 4:**
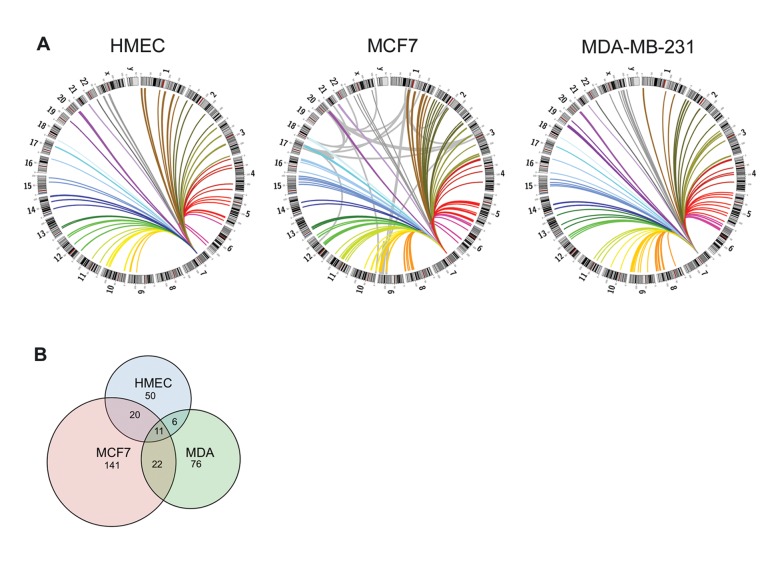
Interchromosomal interaction profile of *IGFBP3*. A, Circos plots showing the distribution of significant interchromosomal interactions involving *IGFBP3* in HMEC, MCF7 and MDA-MB-231. Grey lines in MCF7 plot represent interchromosomal translocations, adapted from Hampton *et al*. [31], falling within windows of significant 4C interactions. B, Venn diagram showing the number of unique and overlapping significant interchromosomal interactions for a window size of 200 consecutive restriction fragments.

We constructed circos plots to highlight the significant interchromosomal interactions involving the *IGFBP3* enhancer in HMEC, MCF7 and MDA-MB-231 that fell within a window size of 200 (Figure 4A, Figure S2). There were a total of 87 significant interactions in HMEC, 194 in MCF7 and 115 in MDA-MB-231. Of these interactions only 11 were common to all samples (Figure 4B, Table 1). Because a large proportion of the significant 4C windows fell within chromosome regions prone to rearrangements, fusions and amplifications, we compared the locations of 157 breakpoints mapped in MCF7 cells [31] to the list of regions that participated in significant interchromosomal interactions. We have limited our analysis to the relationship between interactions in normal HMEC and known breakpoints in MCF7 since it was the only cell line with comprehensive breakpoint data available. This allows for the correlation of interactions pre and post breakage. The MCF7 breakpoints could be categorized as 2 distinct types. The first category contains the majority of breakpoints, which are dispersed throughout the genome in regions of low copy repeats. The second category includes MCF7 breakpoints falling within four highly amplified regions located on chromosomes 1, 3, 17 and 20. We found that breakpoint regions that also participated in interchromosomal interactions were almost exclusively in the latter category. We then considered a subset of 74 MCF7 breakpoints, described as interchromosomal rearrangements, and determined how many were associated with long-range chromatin interactions in HMEC and MCF7 cell lines (Table 2). A total of 29 breakpoint ends mapped within significant windows in HMEC, as compared to 61 in the MCF7 line. All but one of the breakpoints within HMEC 4C windows was also present within MCF7 4C windows. Importantly, when we compared the number of breakpoints for which both ends of the breakpoint mapped to a 4C hit, the percentage was nearly twice as many in the breast cancer cell line MCF7 as in HMEC. This suggests that when an *IGFBP3* interacting region undergoes a translocation involving a different chromosome, the *IGFBP3* interaction is not lost, but instead the translocation brings into proximity an additional interaction detectable by 4C.

**Table 1:** Common *trans* interactions among all samples.

window	genes
chr20:51905176-52752692	*BCAS1, ZNF217,TSHZ2, SUMO1P1, MIR4756*
chr17:58766326-59643391	*BCAS3, TBX2, C17orf82, TBX4*
chr20:48615985-49541607	*BCAS4, LINC00651, UBE2V1, TMEM189,CEBPB, LOC284751, PTPN1, MIR645, FAM65C, PARD6B, ADNP*
chr20:46815739-47725424	*LINC00494, PREX1, ARFGEF2, CSE1L*
chr1:144919188-145816358	*PDE4DIP, SEC22B, NOTCH2NL, NBPF10, HFE2, TXNIP, POLR3GL, ANKRD34A, LIX1L, RBM8A, GNRHR2, PEX11B, ITGA10, ANKRD35, PIAS3, NUDT17, POLR3C, RNF115, CD160, PDZK1, GPR89A*
chr20:45119530-45995741	*ZNF334, OCSTAMP, SLC13A3, TP53RK, SLC2A10, EYA2, MIR3616, ZMYND8, LOC100131496*
chr3:196975652-197787067	*DLG1, MIR4797, DLG1-AS1, BDH1, LOC220729, KIAA0226, MIR922, FYTTD1, LRCH3, IQCG, RPL35A, LMLN, ANKRD18DP*
chr1:200591661-201448561	*DDX59, CAMSAP2, GPR25, C1orf106, KIF21B, CACNA1S, ASCL5, TMEM9, IGFN1, PKP1, TNNT2, LAD1, TNNI1, PHLDA3*
chr2:24898227-25798560	*NCOA1, PTRHD1, CENPO, ADCY3, DNAJC27, DNAJC27-AS1, EFR3B, POMC, DNMT3A, MIR1301, DTNB*
chr4:1134384-2497968	*SPON2, LOC100130872, CTBP1, CTBP1-AS1, MAEA, UVSSA, CRIPAK, FAM53A, SLBP, TMEM129, TACC3, FGFR3, LETM1, WHSC1, SCARNA22, WHSC2, MIR943, C4orf48, NAT8L, POLN, HAUS3, MXD4, MIR4800, ZFYVE28, LOC402160, RNF4*
chr9:132166602-133421900	*LOC100506190, C9orf50, NTMT1, ASB6, PRRX2, PTGES, TOR1B, TOR1A, C9orf78, USP20, FNBP1, GPR107, NCS1, ASS1*

**Table 2:** Distribution of MCF7 translocation breakpoints.

	HMEC	MCF7
4C windows containing at least one breakpoint end	11.5%	13.4%
Total number of breakpoint ends mapping to 4C windows	29	61
Number of breakpoint ends common to HMEC and MCF7	28	28
Breakpoints with both ends in 4C windows	34.5%	68.9%

### Breast Carcinoma Amplified Sequence (BCAS1-4) Genes Interact Significantly with IGFBP3 and Each Other in Normal Breast Cells

**Figure 5:**
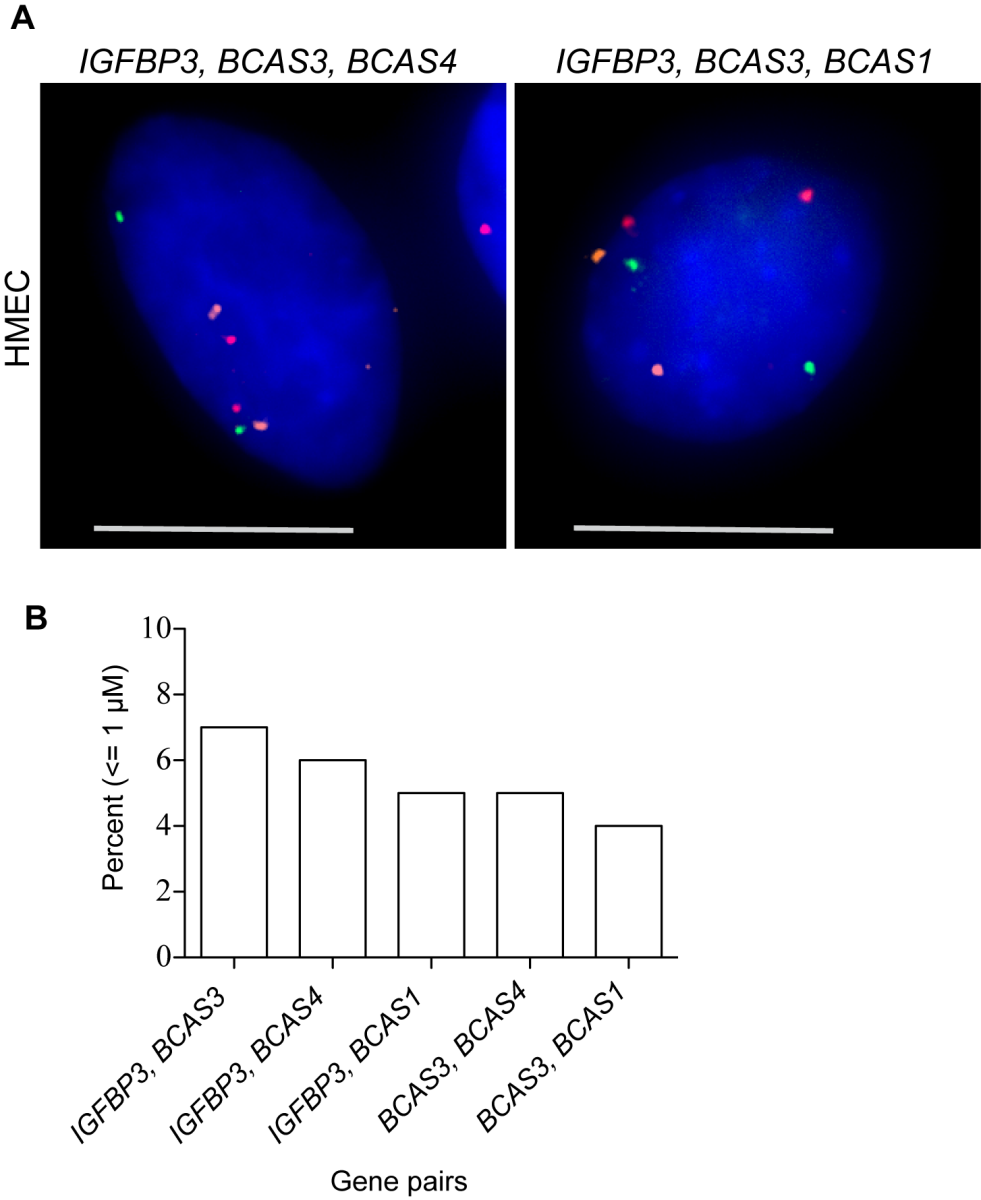
*IGFBP3* interacts with *BCAS* genes. A, Representative triple labeled 3D-FISH, z-axis projection images of *IGFBP3, BCAS3, BCAS4* (left) and *IGFBP3, BCAS3, BCAS1* (right). Scale bar  = 10 µm. B, Percentage of nuclei with the listed pair of gene loci within 1 micron of each other. Distances were measured between the closest two foci in each nucleus.

Some of the most significant 4C-seq interchromosomal interactions in HMEC included regions containing the genes *BCAS 1-4* located on chromosomes 1, 17 and 20. All 4 of these genes were found among the 10 most significantly enriched regions in HMEC, and the region containing *BCAS1* and *ZNF217* was the overall top scoring window. These interactions were also enriched in MCF7, where they are frequently rearranged and amplified (Table 3). We used 3D-FISH to investigate whether the *IGFBP3* interacting *BCAS* genes were also in close spatial proximity with one another prior to any oncogenic translocations (Figure 5). We performed dual and triple labeled 3D-FISH with probes for *IGFBP3*, *BCAS1*, *BCAS3* and *BCAS4* in primary HMEC cells (Figure 5A). Center-to-center distances were measured for the closest pairs of foci for each probe (Figure 5B). All probes targeting the *BCAS* genes were in close proximity, residing less than or equal to 1 micron to *IGFBP3* in at least 5% of nuclei. The *BCAS3-BCAS4* and *BCAS3-BCAS1* regions, which undergo translocations with one another in MCF7 [31], were also within 1 micron in at least 4% of normal HMEC nuclei. These percentages are in line with reports of positive *trans* interacting loci identified using other molecular assays [32], [33]. This suggests spatial proximity of the *BCAS* genes in normal breast cells contributes to their frequent oncogenic translocations.

**Table 3:** *BCAS* gene loci are located in significantly interacting 4C windows.

Cell Line	*BCAS1* chr20	*BCAS2* chr1	*BCAS3* chr17	*BCAS4* chr20
HMEC	1	10	3	5
MCF7	4	1	7	14
MDA-MB-231	1	NA	8	5

Numbers represent rank by p-value with 1 being the most significant interaction.

### Methylated Promoters in Breast Cancer Disproportionally Fall within 4C Windows

Using genome-wide CpG methylation data from Sproul *et al.* [34], we analyzed the distribution of methylated promoters in our 4C data sets. CpG sites with a value equal or greater than 0.8 were considered methylated. Consistent with an increase in global CpG methylation in breast cancer, the total number of methylated sites was greater in MCF7 (3847 sites) and MDA-MB-231 (3282 sites), compared with HMEC (374 sites). There is a significant increase in the proportion of methylated promoters that participated in long-range interactions with *IGFBP3* in both breast cancer cell lines relative to HMEC. This increase was more pronounced in MCF7 cells where *IGFBP3* itself is hypermethylated (Table S2). After correcting for the total number of methylated sites, there was a 3.77-fold (Fisher’s exact test, one-sided p-value 4.742×10^−9^) and 2.85-fold (Fisher’s exact test, one-sided p-value 1.122×10^−5^) increase in methylated promoters located within our 4C windows in MCF7 and MDA-MB-231, respectively.

## Discussion

Chromatin structure plays a key role in establishing and maintaining tissue- specific gene expression profiles throughout development. Epigenetic modification of chromatin can influence DNA packaging and accessibility to *trans* acting regulatory factors. Active regulatory regions are maintained in open chromatin, characterized by nucleosome depletion and DNase I hypersensitivity [35]. A vast number of transcription factor binding sites are situated far from any transcription start site, and interactions occurring among distant regulatory elements can regulate gene expression [30]. Long-range interactions between active regulatory elements may therefore provide a means to fine tune gene activity.

The importance of long-range interactions may be especially relevant in cancer where genomic instability and extensive epigenetic modification of chromatin is common. Rickman *et al.,* for example, found that overexpression of an oncogenic transcription factor in normal cells leads to large-scale changes in chromatin organization [36]. We have seen that there is a dramatic change in long-range interactions in cancer cells compared with cells derived from normal tissues. We have previously shown that loss of IGF2 imprinting in cancer is accompanied by loss of normal long-range intrachromosomal interactions involving the IGF2/H19 locus [11]. In this study we have expanded our view of long-range interactions in cancer by exploring the genome-wide interaction profile of *IGFBP3*.

IGFBP3 plays a major role in IGF signaling through binding the majority of circulating IGF-I and IGF-II, and it may also function independently in a growth stimulating or inhibitory fashion depending on the system studied. We observed that *IGFBP3* interacts with epidermal growth factor receptor (*EGFR)* in all 3 cell lines. EGFR is a receptor tyrosine kinase whose dysregulation can promote tumorigenesis, and nuclear EGFR has been shown to function as a transcription factor to activate genes required for cell proliferation [37], [38]. Recently, the cancer genome atlas network identified four major subtypes of breast cancer based on extensive genomic analyses. They found high level EGFR and phosphorylated EGFR to be associated with a subset of breast cancers with HER2 enrichment, suggesting possible targets for combined therapy [39]. Crosstalk exists between insulin-like growth factor 1 receptor (IGF1R), and other signaling receptors including EGFR. Inhibiting either IGF1R or EGFR results in activation of the reciprocal receptor, suggesting that combined inhibition of both pathways may yield enhanced tumor therapy [40]. There is also interplay between IGFBP3 and EGFR in cancer cells. The initial reaction of ER positive T47D breast cancer cells to IGFBP3 is inhibitory, yet prolonged expression of *IGFBP3* cDNA stimulates growth. Chronic exposure of cells to IGFBP3 over many passages *in vitro* also led to an increase in EGFR protein levels, and enhanced the response to EGF as demonstrated by an increase in both phosphorylated EGFR and DNA synthesis. Furthermore, xenograft tumors in mice that expressed *IGFBP3* showed enhanced growth and increased levels of EGFR [41]. Conversely, overexpression of *EGFR* in primary keratinocytes resulted in 4.4-fold induction of *IGFBP3* [42]. Our 4C-seq and 3D-FISH data indicate *IGFBP3* and *EGFR,* separated by 9 Mb, are often in close spatial proximity (Figure 3). Spatial proximity of loci residing on the same chromosome is influenced to some extent by their linear separation in base pairs; it is therefore difficult to make comparisons between studies of loci with differing amounts of linear separation. Nonetheless, the number of nuclei scored by 3D-FISH containing *IGFBP3* and *EGFR* in close proximity can be considered high in our cell lines, especially HMEC and MDA-MB-231, where nearly all cells had at least one allele demonstrating proximity within 1 micron. Importantly, this high interaction frequency was not due solely to linear distance between the genes, as a large number of interactions occurred monoallelically. This can be observed in HMEC and MDA-MB-231 3D-FISH images in Figure 3A.

Mounting evidence suggests eukaryotic transcription occurs in localized factories [43], [44]. Transcription factories may exist to provide coordinated expression of coregulated genes. By uniting distant regions of DNA they may also serve as sites to share specific or limiting regulatory factors, and may be required for high levels of transcription. We observed that in cell lines with increased *IGFBP3* mRNA there is also an increase in the interaction frequency of *IGFBP3* with *EGFR*. The relationship between interaction frequency and expression is nonlinear, and we expect other factors are modulating expression such as the observed hypermethylation of the *IGFBP3* promoter. Additional factors may include crosstalk between IGFBP3 and EGFR signaling pathways and tumor heterogeneity. Our data suggest that *IGFBP3* and *EGFR* may share a common transcriptional hub or factory, and disruption of these interactions could play a role in tumor progression. Reduction of the *IGFBP3-EGFR* interaction may not only affect these genes, but could result in new long-range interactions.

Cytogenetic and molecular evidence suggests spatial proximity influences recurrent chromosomal translocations [45], [46], [47], [48]. In response to genotoxic stress, oncogenic translocations could potentially form when DNA breaks occur within an interacting “hub”. This was demonstrated in prostate cancer cells where irradiation led to translocations among genes with hormone-induced proximity [49], [50].

From our 4C data, we found that the breast carcinoma amplified sequence family of genes (*BCAS1, BCAS2, BCAS3* and *BCAS4*) interacts with *IGFBP3*. *BCAS1* has been found amplified in primary breast tumors [51] and associated with a poor prognosis [52]. *BCAS2* can function as a transcriptional coactivator of estrogen receptor [53] as well as a negative regulator of *P53* [54]. *BCAS3* is overexpressed and associated with impaired response to tamoxifen in ER positive premenopausal breast cancers [55]. Fine mapping of breakpoints in MCF7 revealed *BCAS3* to be located in a rearrangement hotspot, where 7 breakpoints were observed within *BCAS3* and 19 in the surrounding region of the gene [31]. One of the translocation partners of *BCAS3* is *BCAS4*, and fusion transcripts have been detected in MCF7 and HCT116 colon cancer cells [31], [56]. Additionally, *BCAS4* was found overexpressed in nine out of 13 different breast cancer cell lines [57]. The *BCAS* genes are frequently amplified and some have been found to translocate with each other in breast tumors, such as *BCAS4-BCAS3* and *BCAS1-BCAS3*. Interestingly, using 3D-FISH in normal breast cells, we found *BCAS4- BCAS3* and *BCAS1- BCAS3* to interact with one another as well as *IGFBP3,* supporting the role of spatial proximity in oncogenic translocations. All pairwise interactions, defined as being equal to or less than 1 micron, occurred in 4% or greater of HMEC nuclei. This is similar to the association levels measured for loci participating in interchromosomal interactions identified using the tethered chromosome conformation capture assay [33]. It is also similar to colocalization levels of genes that occupy specialized transcription factories in mouse erythroid nuclei [32]. We chose to verify 4C interactions with 3D-FISH, as the interacting regions can be large, consisting of windows of 100 or 200 restriction sites. 3C would provide better resolution, but doing so on such large regions would be quite challenging considering the number of primers that would be needed since detecting an interaction between two specific elements alone with 3C is not technically sound.

Although all interactions were present within the population of cells, there was not a simultaneous association of all three loci. This suggests the long-range interactions of the *BCAS* genes with *IGFBP3* and with one another are dynamic in nature, and illustrates the heterogeneity of chromatin architecture within a cell population. Chromatin displays rapid constrained motion over distances of ∼ 1 micron and longer directional movement of chromatin domains has been associated with gene expression [58]. We note that 3D-FISH experiments were performed in cycling cells. Since this data is limited to interphase cells we don’t expect it to have a major effect on our results. As the field progresses we will likely see 4D studies incorporating cell cycle stages; there have already been correlations drawn between Hi-C data and replication timing [59].

It remains to be seen what role *trans*-acting factors play in mediating these long-range interactions. In the case of prostate cancer, the androgen receptor was shown to rapidly induce long-range interactions both in *cis* and in *trans* following ligand binding [49], [50]. Estrogen was also shown to induce rapid interchromosomal interactions among estrogen receptor α (ERα) regulated genes [5], [60]. In addition to nuclear receptor mediated long-range interactions, increased expression of the architectural protein SATB1, which participates in chromatin loop formation, alters the expression of over 1000 genes and is associated with aggressive breast cancer [61]. Whatever the mechanism governing long-range interactions, it is likely to involve a combination of chromatin remodeling complexes and possibly nuclear motor proteins. Along these lines, chromatin interacting with *IGFBP3* in the breast cancer cell lines was significantly enriched for methylated promoters relative to HMEC, with MCF7 showing the greatest fold increase. The *IGFBP3* promoter is hypermethylated in MCF7, and this may indicate a preference for chromatin domains with similar modifications to associate.

It is notable that a large proportion of the MCF7 translocation breakpoints fall within 4C windows. To rule out artifacts due to an interaction with a breakpoint near our bait, we checked for breakpoints proximal to *IGFBP3*, but found none within ±5 Mb. It is important to note that MCF7 breakpoints mapped in HMEC reflect areas of potential translocations. It is interesting that all HMEC 4C windows containing translocation breakpoints were also present in MCF7, where breakage had occurred. There was also an increase in breakpoints with both ends mapping to 4C windows in MCF7 as compared to HMEC. In these instances, the *IGFBP3* long-range interactions present in normal breast cells were maintained in the tumor cells, and additional interactions with the reciprocal breakpoints were formed due to rearrangements in the cancer cell line. This indicates a preference for gaining *trans* interactions even after large-scale genomic aberrations occur.

Our study demonstrates that long-range interactions of cancer-related loci, including *EGFR* and *IGFBP3*, are altered in breast cancer cells, and these alterations are frequently associated with epigenetic changes. Long-range interactions influence chromosomal translocations, and add an additional layer of complexity to transcriptional and epigenetic regulation to coordinate gene expression. Therefore, a better understanding of aberrant chromatin interactions is needed to fully understand cancer pathology.

## Methods

### Cell Culture

Primary human mammary epithelial cells, HMEC (Life Technologies, Grand Island, NY) were cultured in HuMEC Ready Medium (Gibco, Grand Island, NY) with 1% penicillin-streptomycin (Gibco). Human breast cancer cell lines MCF7 and MDA-MB-231 (ATCC, Manassas, VA) were grown in Dulbecco’s Modified Eagle Medium (DMEM) with high glucose, sodium pyruvate, GlutaMAX media supplemented with 10% fetal bovine serum, 1% penicillin-streptomycin (Gibco) at 37°C in 5% CO_2_.

### Circular Chromosome Conformation Capture (4C) Sequencing Assay

4C was performed as in Gheldof *et al*. with minor modifications [62]. HMEC, MCF7 and MDA-MB-231 cells (2×10^7^) were fixed in 2% formaldehyde in fresh medium for 10 min at room temperature, followed by quenching with 0.125 M glycine. Fixed cells were scraped from culture plates, spun, (750×g for 10 min), and the frozen pellets were stored at −80°C until lysis. Cells were resuspended in ice-cold lysis buffer (0.2% IGEPAL CA-630, 10 mM NaCl, 10 mM Tris HCl) with SigmaFast complete protease inhibitor tablet (Sigma-Aldrich, St. Louis, MO) and lysed for 30 min on ice. After recovery of nuclei by centrifugation (2000×g for 5 minutes), nuclei were washed twice in cold 1.2× NEB buffer 2 and resuspended in the same buffer. Nuclei were incubated in the presence of 0.3% SDS for 1 h at 37°C with shaking at 950 rpm, followed by the addition of Triton X-100 to 1.8% for 1 h at 37°C with shaking at 950 rpm. Nuclei were digested with 1500 U of HindIII (New England Biolabs Ipswich, MA) overnight at 37°C with shaking at 950 rpm. 200 µl of digested nuclei were removed for assessing digestion efficiency by qPCR. The restriction enzyme was inactivated by the addition of 1.6% SDS and was incubated at 65°C for 20 min. The digested nuclei were diluted in 7 ml of 1.1× T4 DNA ligase buffer in the presence of 1% Triton X-100 and incubated for 1 h at 37°C. Ligation was performed by adding 800 U of T4 DNA Ligase (2,000,000 U/ml; New England Biolabs) to the diluted mixture of digested nuclei and incubating in a 16°C H_2_O bath for 4 hours followed by a 30 min incubation at room temperature. To reverse cross-links, proteinase K was added to a final concentration of 100 µg/ml and incubated overnight at 65°C. Samples were incubated with 0.5 µg/ml of RNase A at 37°C for 1 h and purified by phenol-chloroform extraction followed by ethanol precipitation. DNA concentration was measured using a Qubit® 2.0 Fluorometer (Life Technologies).

3C templates were digested with 200 U MspI (New England Biolabs) overnight at 37°C with shaking at 500 rpm, followed by heat inactivation at 65°C for 20 min. Digestion products were purified by phenol-chloroform extraction and ethanol precipitation. Ligations were performed in 14 ml of 1× T4 DNA ligase buffer with 2000 U of T4 DNA ligase. Circular ligation products were purified by phenol-chloroform extraction and ethanol precipitation followed by clean up with Ampure beads (Beckman Coulter, Brea, CA). A total of 16 inverse PCR reactions with 200 ng input per 4C template were performed for each library with primers that included Illumina adapter sequences and custom barcodes. All PCR reactions were performed with Expand Long Template PCR system (Roche, Indianapolis, IN). Excess primers were removed by gel extraction. HMEC, MCF7 and MDA-MB-231 4C libraries were analyzed on a MultiNA microchip electrophoresis system (Shimadzu Columbia, MD) and mixed in equimolar amounts. Multiplex sequencing was performed on an Illumina genome analyzer IIx (Illumina, San Diego, CA). Illumina sequencing data have been submitted to the GEO database accession number: GSE49521.

### Mapping and Filtering of 4C Reads

We first de-multiplexed the 76 bp single-end reads using barcodes for each cell line. We only retained the reads that contained one of the valid barcodes followed by the primer sequence and a HindIII cleavage site and truncated them to obtain the prey sequence. We mapped the truncated reads to the human genome (UCSC hg19) using the short read alignment mode of BWA (v0.5.9) with default parameter settings. We post-processed the alignment results to extract the reads that satisfied the following three criteria: (i) mapped uniquely to one location in the reference genome, (ii) mapped with an alignment quality score of at least 30 (which corresponds to 1 in 1000 chance that mapping is incorrect), (iii) mapped with an edit distance of at most 3. We assigned the qualified reads to the nearest HindIII cleavage site using their mapping coordinates. We then identified the restriction fragments interacting (those flanking the cleavage sites with a read count of at least one) with the bait region. We discarded ±50 kb region around the bait from further analysis.

### Statistical Analysis of 4C Data

We first identified all the HindIII sites in the genome (∼840 k) and eliminated the ones with no MspI site within 2 kb downstream of the HindIII site, resulting in ∼470 k restriction fragments for downstream analysis. In order to avoid PCR artifacts, we binarized the interactions counts as was done previously in other 4C analysis pipelines [63]. This processing resulted in 23,559, 19,876 and 16,387 restriction fragments that interact with the bait region for HMEC, MCF7 and MDA-MB-231 cell lines, respectively. In order to account for the difference in the number of interacting fragments between cell types and the effect of genomic distance on the intrachromosomal interaction probability, we applied a statistical significance assignment procedure similar to the one described in Splinter *et al* [63]. We first separated interactions into four groups depending on the linear distance of interacting loci to the bait.

1. Bait region interactions: Intrachromosomal interactions below 50 kb distance to the bait and are excluded from our analysis.
2. Proximal intrachromosomal interactions: Intrachromosomal interactions between 50 kb to 2 Mb distance from the bait.
3. Long-range intrachromosomal interactions: Intrachromosomal interactions above 2 Mb distance from the bait.
4. Interchromosomal interactions: Interactions that are on chromosomes other than the bait chromosome (chr 7).

We then combined multiple consecutive restriction fragments with window sizes that are appropriate for each of the groups above. This step is necessary due to limited resolution of current 4C methods and enables us to assign statistical confidences for interactions at varying resolutions. We used window sizes of 10, 20 and 40 for group 2; 50, 100 and 200 for group 3; 100, 200 and 400 for group 4 interactions. For each group of interactions, we counted the number of interacting fragments within a window for each window size. We then generated a background distribution by randomly shuffling the interacting and non-interacting fragments for each group and repeating this randomization 100 times. For intrachromosomal interactions, we take into account the linear distance of each region to the bait when generating the background. For interchromosomal interactions, we generated the background by aggregating all chromosomes (unlike Splinter et al [63] who generate one background per each chromosome) to preserve the information from possible chromosome territory associations that include chromosome 7. Similar to Splinter *et al*, [63] we calculated the z-value threshold at which the false discovery rate (FDR) is 0.01 to determine the windows that significantly interact with the 4C bait (4C-enriched windows/regions). To determine cell line specific 4C-enriched regions, at a given window size, we simply take the list of regions that are deemed interacting at FDR 0.01 in one cell line and not in the other.

### 3D-fluorescence *in situ* Hybridization

Cells grown on 12 mm coverslips were fixed in 4% paraformaldehyde (PFA) for 10 min, made permeable with 0.5% Triton X-100 for 5 min, incubated in 20% glycerol/1× PBS for at least 40 min, freeze-thawed in liquid nitrogen four times, and treated with 0.1 N HCl for 5 min. Cells were then treated with RNase A for 45 min at 37°C. Coverslips were then stored in 50% formamide/2× SSC at 4°C until denaturation at 75°C for 7 min in 70% formamide/2× SSC followed by immersion in ice cold 50% formamide/2× SSC.

BAC probes: RP11-89E8, RP11-1083I7, RP11-55E1, RP11-1115J10, RP11-705A3, RP11-805G4, RP11-185P21, RP11-1058F18, RP11-937E18, RP11-5P14 (Roswell Park Cancer Institute, Buffalo, NY) were labeled with dinitrophenol-11-dUTP (PerkinElmer, Waltham, MA), Alexa488-dUTP or Alexa594-dUTP (Life Technologies) by nick-translation (Roche). Probes in 50% formamide/2× SSC/10% dextran sulfate were denatured for 8–10 min at 75°C. Probes were cooled on ice and hybridized for 36-48 h at 37°C, followed by three post-hybridization washes with 50% formamide/2× SSC/0.05% Tween 20, 2× SSC/0.05% Tween 20, and 1× SSC for 30 min each at 37°C. Detection of BAC probes was performed by reaction with rabbit anti-DNP (Life Technologies) diluted (1∶1000) and secondary goat anti-rabbit (1∶200) conjugated to Alexa594 or Alexa647 (Life Technologies). Following labeling, indirect immunofluorescence was detected with Chroma filter sets using an Olympus BX41 upright microscope (100× UPLSAPO, oil, 1.4 NA) equipped with motorized *z*-axis controller (Prior Scientific, Rockland, MA) and Slidebook 5.0 software (Intelligent Imaging Innovations, Denver, CO). Optical sections of 0.5 µm were collected, deconvolved using a NoNeighbor algorithm operating within Slidebook 5.0, and 3D distances were measured from the center of each FISH focus.

### CpG Methylation by Bisulfite Pyrosequencing

Genomic DNA from HMEC, MCF7 and MDA-MB-231 were treated with bisulfite using the EZ DNA Methylation kit (ZYMO Research, Irvine, CA). The locus of interest was amplified using a combination of forward and biotinylated reverse primers (see Table S3 for primer sequences). 40 ng bisulfite-treated DNA was used for each 25 µl PCR reaction with 2G Robust polymerase (KAPA Biosystems, Woburn, MA) following KAPA’s recommended cycling conditions. Pyrosequencing of the resulting amplicons was performed at the PAN facility, Stanford University using a Qiagen Pyromark instrument. Assays were designed using Pyromark Assay Design software (Qiagen, Valencia, CA). The methylation indices were calculated as the average percent methylation of successive CpG dinucleotides between the primers.

### RNA Extraction and Quantitative RT-PCR

RNA was extracted from HMEC, MCF7 and MDA-MB-231 cells using the RNeasy Mini Kit and QIAshredder mini column (Qiagen) according to the manufacturer’s instructions. DNA was digested on a column using RNase free DNase set (Qiagen). 1 µg of RNA was reverse transcribed with Superscript III first-strand synthesis supermix for qRT-PCR (Life Technologies). qRT-PCR was performed using KAPA SYBR Fast ABI PRISM qPCR mix (KAPA) on an ABI 7900HT Real-Time PCR System (Applied Biosystems). Primers were purchased from RealTimePrimers.com. The most stable reference genes (*ACTB* and *GAPD*) were selected from a set of 10 using geNorm software [64]. Reaction efficiency for each primer set was calculated using Real-time PCR Miner [65] and fold change of target genes relative to HMEC was calculated using the Pfaffl method [66].

## Supporting Information

Figure S1 ***IGFBP3*** **4C-Seq Bait.** The bait sequence, top (red bar) flanks a HindIII site upstream of *IGFBP3* in a region classified as a strong enhancer (orange bar). Image generated with UCSC genome browser, hg19.
(JPG)

Figure S2 **Distribution of the significant 200 restriction site interchromosomal windows for HMEC, MCF7 and MDA-MB-231.** Percent of total interactions per cell line are plotted for each chromosome.
(JPG)

Table S1 Sequence read distribution (not corrected for local interactions).
(JPG)

Table S2 Distribution of methylated promoter CpG nucleotides relative to HMEC.
(DOCX)

Table S3 Methylation assay primer sequences.
(DOCX)
