# Supplementary figures and images for: Genomic Interaction Profiles in Breast Cancer Reveal Altered Chromatin Architecture
Source: PLoS One. 2013 Sep 3;8(9):e73974. doi: 10.1371/journal.pone.0073974 (PMC3760796; doi:10.1371/journal.pone.0073974)

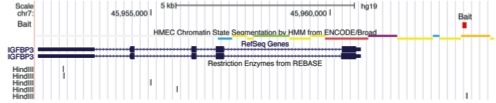

Supplement: Figure S1 — IGFBP3 4C-Seq Bait. The bait sequence, top (red bar) flanks a HindIII site upstream of IGFBP3 in a region classified as a strong enhancer (orange bar). Image generated with UCSC genome browser, hg19. (JPG) [file pone.0073974.s001.jpg]

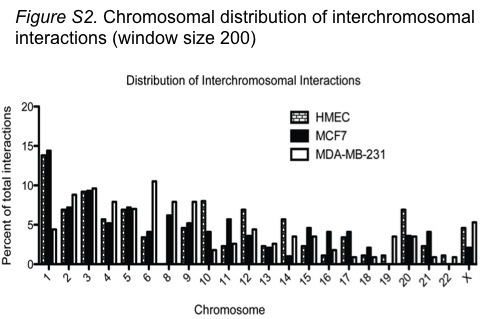

Supplement: Figure S2 — Distribution of the significant 200 restriction site interchromosomal windows for HMEC, MCF7 and MDA-MB-231. Percent of total interactions per cell line are plotted for each chromosome. (JPG) [file pone.0073974.s002.jpg]

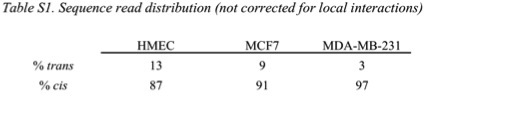

Supplement: Table S1 — Sequence read distribution (not corrected for local interactions). (JPG) [file pone.0073974.s003.jpg]
