# Supplementary material for: Genomic Interaction Profiles in Breast Cancer Reveal Altered Chromatin Architecture
Source: PLoS One. 2013 Sep 3;8(9):e73974. doi: 10.1371/journal.pone.0073974 (PMC3760796; doi:10.1371/journal.pone.0073974)
Supplement: Table S2 — Distribution of methylated promoter CpG nucleotides relative to HMEC. (DOCX) [file pone.0073974.s004.docx]

| Threshold(≥0.8) | methylated in 4C windows (fold change) | methylated outside 4C windows (fold change) | corrected methylated in 4C windows (fold change) |
| --- | --- | --- | --- |
| MCF7 | 38.79 | 9.18 | 3.77 |
| MDA-MB-231 | 25.00 | 8.14 | 2.85 |

**Table S2**. distribution of methylated promoter CpG nucleotides relative to HMEC
