# Supplementary material for: Genomic Interaction Profiles in Breast Cancer Reveal Altered Chromatin Architecture
Source: PLoS One. 2013 Sep 3;8(9):e73974. doi: 10.1371/journal.pone.0073974 (PMC3760796; doi:10.1371/journal.pone.0073974)
Supplement: Table S3 — Methylation assay primer sequences. (DOCX) [file pone.0073974.s005.docx]

*Table S3. methylation assay primer sequences*

| Locus | Forward | Reverse (5' biotin) | Sequencing | T_A_ (°C) |
| --- | --- | --- | --- | --- |
| ***IGFBP3*P1** | GTTTTAGGGGTGAGGTTTTTTT | ACCCCCCAATACCTTAACTCCCT | GGTGTAGGGGAAATTGG | 57 |
| ***EGFR*P1** | GGTTTTGGAGGAAAAGAAAGGTA | TTCCCCCCCTAACCTACAACCC | GGAGGAAAAGAAAGGTAA | 59 |
